# The association between cell assemblies and transient dynamics

**DOI:** 10.1186/1471-2202-15-S1-P10

**Published:** 2014-07-21

**Authors:** Christian Tetzlaff, Sakyasingha Dasgupta, Florentin Wörgötter

**Affiliations:** 1 III. Institute for Physics - Biophysics, Georg-August University, Göttingen, Germany; 2 Bernstein Center for Computational Neuroscience, Göttingen, Germany

## 

Neural systems show a wide variety of complex dynamics on different time scales. Specifically, on the short time scale of neuronal activations (milliseconds to seconds), several theoretical models (e.g., state-dependent networks or liquid state machines [1]) demonstrate that complex (transient) dynamics enables neural circuits to process a broad range of nonlinear problems. In other words, neural circuits process inputs to transform them into desired outputs. This approach, amongst others, is based on the inherent stochasticity (randomness) of neural systems. On the other hand, the slower mechanisms of synaptic plasticity (minutes to hours) adapt synaptic efficacies to form ordered structures as neural cell assemblies [2]. Each cell assembly consists of a group of strongly interconnected neurons and its dynamics serves as an associative memory of the previously experienced input. This type of dynamics is in stark contrast to the needed randomness of connectivity for transient dynamics. Intriguingly, several experiments show that both concepts coexist in the same neural circuits [3,4].

**Figure 1:**
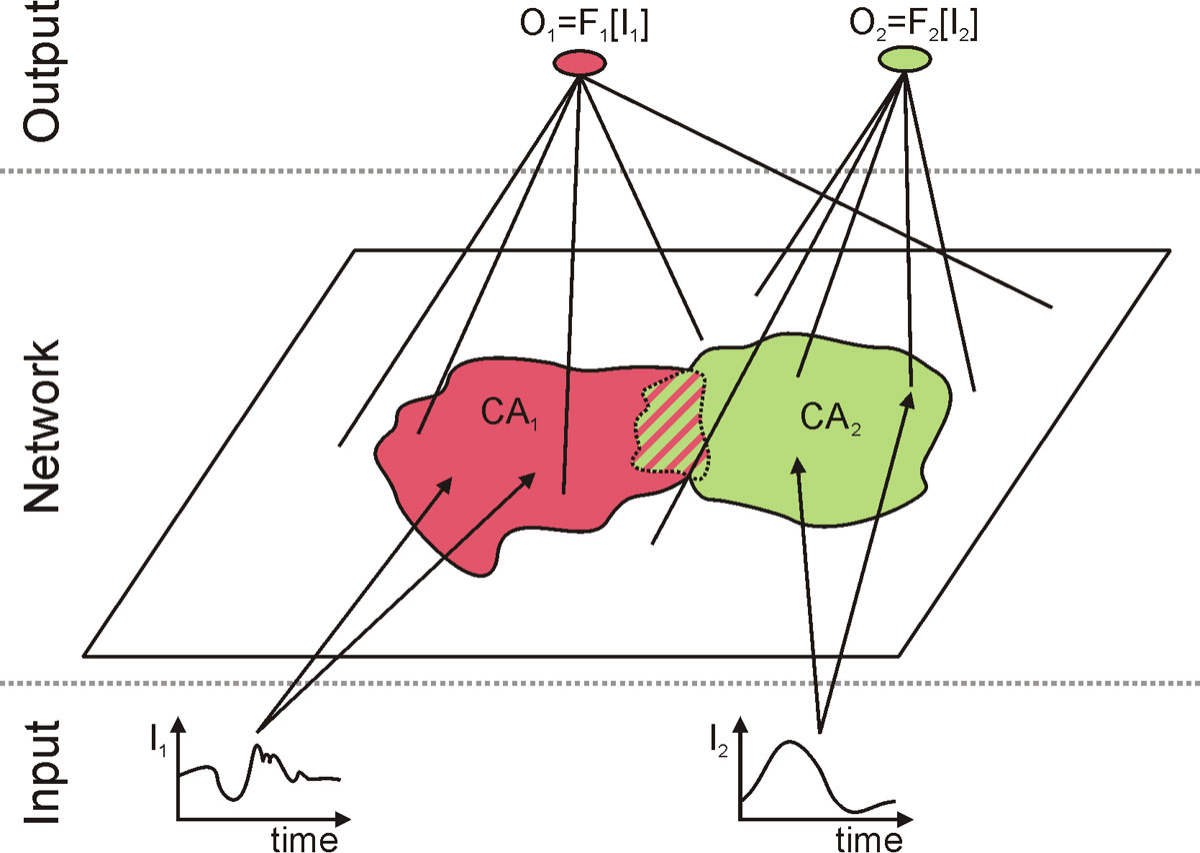
Basic setting of the system. CAi: Cell Assembly i; Ii: Input to CAi; Oi: Output of CAi; Fi: Function processed by CAi

In this study we analyze how this coexistence of transient dynamics and cell assemblies can emerge in one neural circuit. The neural network, we investigate, consists of rate- based neurons with connections adapted by a generic combination of Hebbian plasticity and synaptic scaling [5]. A subset of neurons receives repeatedly a time-varying input and forms a cell assembly (Figure 1). Further repetitions of the input lead to a gradual growth of the cell assembly by incorporating more neurons. In parallel, the network is required to compute a desired nonlinear version of the input. To test whether this computation is successful, the connections between the neurons of the network and a linear output neuron are adapted by a supervised learning algorithm [6]. Remarkably, the performance of the computation increases with the growth in size of the cell assembly. Thus, the slow, ordering dynamics of synaptic plasticity supports the computational capability of the fast transient dynamics. Furthermore, as the cell assemblies are topologically compact, the network is able to perform several computations in parallel (green and red cell assemblies in Figure 1). However, if the cell assemblies become neighbors, dependent on the input, one cell assembly can capture neurons from the other assembly (stripped area in Figure 1). Thus, the cell assemblies compete for computational resources, namely neurons and synapses. Therefore, our work shows a new functional role of cell assemblies in neuronal systems, whereby both stable and transient dynamics are byproduct of the same neural circuit. Thus, both concepts are not mutually exclusive but that they interact with each other in a positive way.
